# Visualization of Customized Convolutional Neural Network for Natural Language Recognition

**DOI:** 10.3390/s22082881

**Published:** 2022-04-08

**Authors:** Tajinder Pal Singh, Sheifali Gupta, Meenu Garg, Deepali Gupta, Abdullah Alharbi, Hashem Alyami, Divya Anand, Arturo Ortega-Mansilla, Nitin Goyal

**Affiliations:** 1 Chitkara College of Applied Engineering, Chitkara University, Chandigarh 140401, Punjab, India; tajinderpal.singh@chitkarauniversity.edu.in; 2 Chitkara University Institute of Engineering and Technology, Chitkara University, Chandigarh 140401, Punjab, India; sheifali.gupta@chitkara.edu.in (S.G.); meenu.garg@chitkara.edu.in (M.G.); deepali.gupta@chitkara.edu.in (D.G.); 3 Department of Information Technology, College of Computers and Information Technology, Taif University, 11099, Taif 21944, Saudi Arabia; amharbi@tu.edu.sa; 4 Department of Computer Science, College of Computers and Information Technology, Taif University, 11099, Taif 21944, Saudi Arabia; hyami@tu.edu.sa; 5 Department of Computer Science, Lovely Professional University, Jalandhar 144401, Punjab, India; divyaanand.y@gmail.com; 6 Higher Polytechnic School, Universidad Europea del Atlántico, C/Isabel Torres 21, 39011 Santander, Spain; arturo.ortega@uneatlantico.es; 7 Department of Project Management, Universidad Internacional Iberoamericana, Campeche 24560, Mexico

**Keywords:** Gurumukhi script, word recognition, convolutional neural network, performance analysis

## Abstract

For analytical approach-based word recognition techniques, the task of segmenting the word into individual characters is a big challenge, specifically for cursive handwriting. For this, a holistic approach can be a better option, wherein the entire word is passed to an appropriate recognizer. Gurumukhi script is a complex script for which a holistic approach can be proposed for offline handwritten word recognition. In this paper, the authors propose a Convolutional Neural Network-based architecture for recognition of the Gurumukhi month names. The architecture is designed with five convolutional layers and three pooling layers. The authors also prepared a dataset of 24,000 images, each with a size of 50 × 50. The dataset was collected from 500 distinct writers of different age groups and professions. The proposed method achieved training and validation accuracies of about 97.03% and 99.50%, respectively for the proposed dataset.

## 1. Introduction

The digitization of printed or handwritten paper documents is performed via text analysis and recognition using a machine, and plays an important role in saving damaged literary, mythological and journal books, etc., for which manual text recognition is impossible. In text analysis and recognition, word recognition is an emerging field. Handwritten word recognition is a method of automatic recognition of handwritten words by a machine. Handwritten word recognition can be performed in two different ways: online handwritten word recognition systems and offline handwritten word recognition systems. In online handwritten word recognition, the words are written using a touch pad and a stylus, and the stylus tip direction is tracked to recognize the word [1]. On the other hand, in offline handwritten word recognition systems, the words are written on an offline document (text written on paper) that is converted into a scanned image for recognition purposes. Furthermore, offline handwritten word recognition can be performed using two different approaches: the analytical approach and the holistic approach.

For word recognition, in an analytical approach, a word is not considered as a single unit. In this approach, firstly a word is split into individual character images before recognition that is known as word segmentation [2]. After word segmentation, several sub-images for each character from a single word image are produced. These characters’ images are recognized separately in order to achieve complete word recognition. Thus, the word recognition result of this approach is the composition of the individually recognized parts. The quality of the sub-images depends upon the method employed for segmentation. However, to achieve an acceptable recognition rate, sub-images should be produced in some meaningful form after segmentation, such that they are easy to process and evaluate. Segmented images are then further processed for feature extraction. After that, based on these features, classification techniques are applied for final reorganization. Often, overlapping characters appear in handwritten text, such as in cursive handwriting, causing problems with segmentation. This problem can be avoided by using a segmentation-free technique for word recognition known as the holistic approach. In this approach, a word is considered as an inseparable unit [3]. This means that no segmentation is performed, and the whole word is recognized at once. This is an alternative method for word recognition in such cases, where it is difficult to find segmentation points, as in the case of cursive handwriting, words with overlapped and touching characters, etc. The feature descriptor in the holistic approach is used to extract the contour or shape information from the word image, leading to its use for object discrimination during the recognition process.

The Gurumukhi script is a complex script. In the Gurumukhi script, the characters usually overlap, and it is very difficult to perform segmentation on it. In this case, recognizing each of the segmented characters individually and combining those characters for the final output also increases the risk of misinterpretation of the script. In recent years, the holistic approach to word recognition has attracted the attention of many researchers in text recognition due to its better results than segmentation-based techniques. Hence, in the present work, a holistic approach to word recognition is proposed for recognition of Gurumukhi month names. So far, no work on offline handwritten Gurumukhi month name recognition using the holistic approach has been reported. The proposed design will be helpful as an automated system for month name recognition in regional applications like recognizing the month on birth or death certificates, billing receipts, etc. The major contributions of this article are:

1. The dataset for the proposed research work was prepared for 24 different classes of Gurumukhi months from 500 different writers of different age groups and professions, where each writer wrote each word twice, resulting in 24,000 words in the Gurumukhi month name dataset.
2. A Convolutional Neural Network is proposed for the prepared Gurumukhi month name dataset.
3. The focus of this work is to examine the overall results of using the Convolutional Neural Network on the prepared dataset in terms of accuracy, precision, recall and F1 score.
4. The proposed model’s performance is evaluated using different numbers of epochs and batch sizes in a comparative performance analysis.
5. A performance comparison is conducted for the proposed Convolutional Neural Network model with various transfer learning models.

To improve overall network performance and results, various image processing operations are performed using neural networks. These operations include reconstruction of the images, image resizing, image segmentation, image scoring, noise removal from images, extraction of regions of interest from images, etc. These operations lead to reduced overall training time of neural networks and improved interpretation of their results. The most popular methods for image processing using neural networks focus on two operational processes. First, the classic architecture is made up of neurons that analyze specific pixels as input and are trained using various algorithms such as back propagation or the heuristic approach, which have become more popular in recent years. For example, the author in [4] reconstructed brain MRI images using a heuristic validation mechanism. The experiment was performed on both the whole image and the small region of interest extracted using a heuristic approach. The results of the article showed that the training time of the network was successfully reduced using the proposed approach. In [5], the researchers performed image compression using attentional multi-scale back projection and frequency decomposition. In this article, the authors provided an optimal solution for preserving spatial information in images by using low-level compression techniques. With this objective, the author developed a novel back projection technique, a novel dual attention module for recombining the distinct frequency components of an image, and a novel training method for reducing the latent rounding residual. The proposed method showed significant improvement over the existing model in terms of preserving spatial information with increased compression quality.

In contrast to image processing using a neural network with back propagation or heuristic approaches, a convolutional neural network is another type of neural model that includes two additional layers devoted to image processing. One is the convolutional layer and the second is the pooling layer. Both are used for feature extraction and image resizing. The same technique was used in the present work on 24,000 digital images of handwritten Gurumukhi month names for the purposes of feature extraction and image resizing.

## 2. Literature Review

In this section, the previous work reported in the field of text recognition in Indian scripts is presented. This literature review section is split into two parts. The first part will discuss the work done on text recognition based on an analytical approach and the second part will discuss the work done on text recognition based on a holistic approach.

In one analytical approach, for example, the researchers proposed two stages of recognition of Online Handwritten Gurumukhi Characters [6]. In the first stage, the authors recognized unknown strokes, while in the second stage, they evaluated the characters on the basis of the strokes recognized in the first stage. As a result, the authors achieved a recognition accuracy of up to 90.08%. Some authors rearranged and identified dependent and major dependent strokes on the basis of their position in order to achieve character recognition in Online Handwritten Gurumukhi Words [7]. The proposed method achieved an 81.02% accuracy rate. A variable size windowing technique has also been used to interpret characters in Gurumukhi text [8]. The proposed technique achieved a segmentation accuracy of 93.3%. Zernike, Pseudo Zernike and orthogonal Fourier–Mellin moments have been applied on bilingual characters (Gurumukhi and Roman) for feature extraction [9]. Of these moments, the Pseudo Zernike moments gave the best result. A review on optical character recognition in various handwritten Indian scripts, including Gurumukhi, Devanagari, Oriya, Tamil, Kannada, etc., has also been presented [10]. It was concluded that the handwriting of various writers can be compared using K-NN, HMM and Bayesian classifiers with the zoning, directional and diagonal features extraction techniques [11]. The researchers performed this experiment on a dataset of Gurumukhi characters collected from 100 different writers and tested 25 different writers’ handwriting in order to grade them on the basis of recognition accuracy. One article [12] demonstrated that different feature extraction techniques like parabola curve fitting and power curve fitting could be used for the recognition of handwritten Gurumukhi characters. The results section of that article concluded that by using curve feature extraction techniques on Gurumukhi characters, an accuracy of up to 98.10% could be achieved when classification was performed using K-NN and SVM classification techniques. Recognition of Gurumukhi Aksharas based on multiple strokes using the RBF-based SVM classifier has also been proposed [13]. The proposed method was evaluated on 4310 handwritten Gurumukhi Aksharas obtained from various users, resulting in a Gurumukhi Mukta accuracy of 93.33%. A one and stroke identification method has been proposed for Gurumukhi text recognition [14]. In that work, the author identified three zones—the upper, middle, and lower zones—in Gurumukhi characters. The authors tested the proposed method on a 428-character dataset written by 10 different writers and achieved 95.3% accuracy in zone identification and 74.8% accuracy in stroke recognition using the SVM classification technique. An offline handwritten Gurumukhi character recognition method has been proposed based on different transform techniques like discrete wavelet transform (DWT2), discrete cosine transform (DCT2), fast Fourier transform and fan beam transform [15]. The proposed method achieved 95.8% accuracy on a testing dataset of 10,500 Gurumukhi characters using the DCT2 feature extraction technique and SVM classification. A zone identification-based algorithm for stroke classification has been proposed in which are classified into two different zones, upper and lower, for the online Gurumukhi characters with Matars [16]. The proposed algorithm achieved zone identification accuracy of up to 99.75% and character recognition accuracy of 97.1% when tested on 21,500 characters that had been collected from 10 different writers. Recognition accuracy of up to 99.3% was achieved on a dataset of 2700 images of offline Gurumukhi characters using a deep neural network [17]. The various feature extraction techniques used in this research work include local binary pattern (LBP) features, directional features, and regional features. Researchers have proposed an algorithm based on finite state automata for the structuring of Gurumukhi characters [18]. The proposed method achieved an accuracy of up to 97.3% for Gurumukhi character formation when tested on 8200 characters written by 20 different authors. Histogram Oriented Gradient (HOG) and Pyramid Histogram Oriented Gradient (PHOG) features have been explored for the recognition of offline handwritten Gurumukhi characters [19]. The simulation results revealed a character recognition accuracy of 99.1% with the SVM classifier for the PHOG feature. A unique approach for writer identification based on the recognition of individual handwritten Gurumukhi characters has also been presented [20]. An accuracy of 89.85% was achieved when using this approach with a combination of zoning-, transition-, and peak-extent-based feature extraction techniques and SVM classification. The dataset for the proposed work contained 31,500 sample images of Gurumukhi characters. According to [21], using various feature extraction techniques like zoning, transition, diagonal, intersection, and open end points, the horizontal peak extent, centroid, the vertical peak extent, parabola curve fitting and power curve fitting, as well as employing classifiers like naive bayes, decision tree, random forest, and AdaBoostM1, writer identification can be done in the context of handwritten Gurumukhi text. The results of this article show that, compared to the combination of classifiers with a combination of other features, the AdaBoostM1 ensemble classifier with centroid features gives a maximum identification accuracy of up to 81.75%. Boosting and Bagging techniques have been proposed for the recognition of handwritten medieval Gurumukhi text [22]. The proposed methodology provided a recognition accuracy of up to 95.91% when compared with other techniques, and used a combination of classifiers with a voting scheme. An unprocessed, unconstrained offline handwritten Gurumukhi character recognition system was devised and applied to 56 different classes of characters in Ref. [23]. In this work, three classifiers, k-NN, decision tree, and random forest, were used for character classification. A maximum recognition accuracy of up to 96.03% was achieved by using a random forest classifier with zoning and shadow features along with a fivefold cross validation technique. In Ref. [24], the authors examined the impact of a combination of feature extraction and classification techniques on the recognition of handwritten Gurumukhi characters. For this experiment, k-NN and SVM classification techniques were used. The results showed that the combination of linear-SVM, polynomial-SVM and k-NN classifiers was able to achieve a recognition accuracy of up to 92.3%. In Ref. [25], an evaluation of the effectiveness of classifiers for the recognition of offline handwritten Gurumukhi characters and numerals was performed. The results indicated that the random forest classifier performed better than the other classifiers when tested on the 13,000 sample images, and giving an accuracy of up to 87.9%. A technique based on a deep convolutional neural network was applied to a dataset of 3500 Gurumukhi characters [26]. With two convolutional and two pooling layers, the network achieved an accuracy of 98.32% on the training set and 74.66% on the test set.

In one holistic approach Hindi word recognition was tested in different states of India using an 89-element feature vector and various classifiers, including MLP, Sequential Minimal Optimization, Logistic Regression Model, Naïve Bayes, and a Multiclass classifier [27]. The results of this experiment showed that MLP outperformed the other classifiers, with an accuracy of up to 96.82%. In Ref. [28], the authors proposed offline Bangla word recognition based on a holistic approach. The proposed design was tested on 18,000 images of handwritten Bangla words using histogram-based features and two classifiers: a multi-layer perceptron (MLP) and a support vector machine (SVM). The results of this experiment showed that SVM outperformed MLP, with an accuracy of up to 83.64%. An eXtreme Gradient Boosting approach for Gurumukhi word recognition has been proposed [29]. This method was tested using a public Gurumukhi script benchmark dataset consisting of 40,000 instances of handwritten words. The effectiveness of the proposed system was validated by the authors on the basis of various parameters, including accuracy (91.66%), F1 score (91.14%), precision (91.39%), recall (91.66%) and AUC (95.66%), using 90% of the dataset for training and 10% of the dataset for testing.

This literature review led us to conclude that, so far, the majority of the research reported on Gurumukhi text recognition has emphasized character recognition, either on isolated characters or on individual characters. Few researchers have worked on Gurumukhi word recognition, and that work which has been performed has been based on traditional methods of text recognition like manual feature extraction and word-to-character segmentation. Elastic matching, K-NN, H-MM, K-NN, state vector machines with kernels (such as linear, polynomial and radial base function) are the various classifiers that have been used in previous work for Gurumukhi text recognition. Deep neural networks have only been used for character recognition. Finally, the method proposed in this work of offline handwritten Gurumukhi month name recognition using a holistic approach by means of a convolutional neural network is novel and has not been reported yet.

The following sections ion this article provide a full description of the proposed CNN model for word recognition of Gurumukhi month names and of the prepared dataset on which the proposed model was validated.

## 3. Dataset Preparation

**Figure 1:**
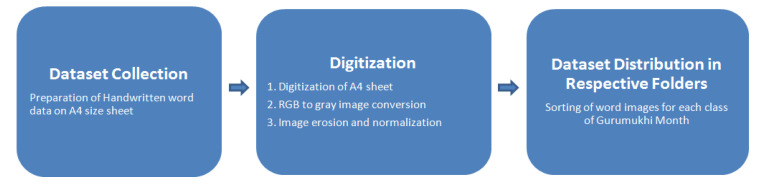
Various steps of dataset preparation.

A dataset was prepared to train and validate the proposed model for Gurumukhi month name recognition, which included various steps as shown in Figure 1.

### 3.1. Dataset Collection

**Figure 2:**
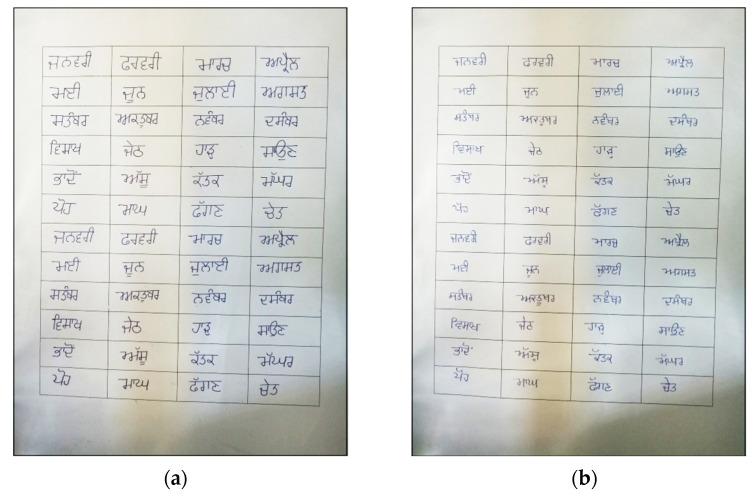
(**a**) Sample sheet from writer 1; (**b**) sample sheet from writer 2.

An offline dataset of handwritten Gurumukhi month names was created on an A4 sheet of paper. This A4 sheet of paper contained handwritten data for 24 different classes of Gurumukhi months written in different blocks drawn on the same sheet. Each block on the sheet had a single handwritten word that belonged to only one class of Gurumukhi month. In total, 1000 words or samples for each class in the 24 different classes of Gurumukhi months were collected from 500 different writers, where each writer wrote each word twice, resulting in 24,000 words or samples in the Gurumukhi month name dataset. For the given dataset, writers from different age groups, genders, and professions were considered in order to provide extreme syntactic variations in the dataset. The sample sheets collected from two different writers are shown in Figure 2a,b. In the sample sheet, each writer wrote each class name two times in the different blocks drawn on the sheet. As a result, 48 handwritten words are written on a single sample sheet for the 24 classes of the Gurumukhi months.

A detailed overview of the prepared dataset is given in Table 1. As per the table, there are twelve months in the Gurumukhi script, and these are known as the Desi months. The names of all the Desi months, as well as well as their corresponding dates on the English calendar, are presented in Table 1.

It is worth noting that Desi months do not begin on the first day of the English month. They usually start in the middle of the English month and end in the same way. For example, the Desi month ‘Vaisakh’ (ਵਿਸਾਖ) starts on the fourteenth (14th) of ‘April’ and ends on the fifteenth (15th) of ‘May’ when considered in English months. The total number of days in ‘Vaisakh’ is thirty-one. In the same way, ‘Bhado’ (ਭਾਦੋਂ) starts on the sixteenth (16th) of ‘August’ and ends on the fourteenth (14th) of ‘September’. ‘Bhado’ has a total of thirty days. The same pattern holds true for all of the Desi months.

Another point to note is that the beginning of the according to the Desi months is different from in English months. ‘Vaisakh’ (ਵਿਸਾਖ) is considered to be the first month of the Desi year, having thirty days. The following months in the Desi year are ‘Jeth’, ‘Harh’, ‘Sawan’, ‘Bhado’, Assu’, ‘Katak’, ‘Magar’, ‘Poh’, ‘Magh’ and ‘Chet’.

English month names written in Punjabi are usually used in Gurumukhi text. Table 1 also shows the English month names alongside their Punjabi translations. English month names written in the Punjabi language have the same number of days as in the English months. For ease of recognition, in this work, we employed both types of month name (Desi months and English month names written in Punjabi).

### 3.2. Digitization

The second step was to convert a collected dataset of Gurumukhi months written on a paper document into a digital format or image. The digitization of paper documents was performed using the 13-megapixel rear camera of an OPPO F1s smart phone. Each digitized image of a paper document had a size of 1024 × 786 pixels. An example of a paper document converted into a digital image is shown in Figure 2a,b. All digitized paper documents were then stored on the local drive of a personal computer.

**Figure 3:**
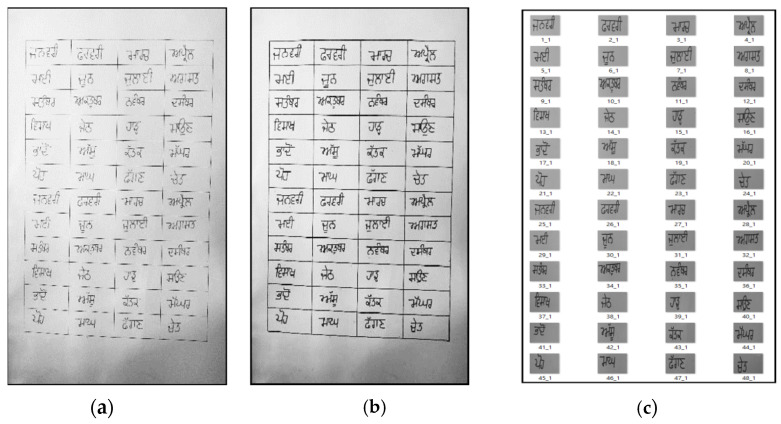
(**a**) Image converted from RGB to grayscale; (**b**) eroded image; (**c**) cropped images.

Image pre-processing was performed on digitized paper documents. During image pre-processing, image conversion from RGB to grayscale, image erosion, and normalization were performed. An image converted from RGB to grayscale is shown in Figure 3a. After that, image erosion was applied to the grayscale images. The objective of image erosion is to make the black sections in the image thicker, as shown in Figure 3b. After image erosion, the grayscale image containing the names of all the Gurumukhi months was cropped into 48 images, with two images for each Gurumukhi month name. The 48 cropped 48 images for the Gurumukhi months are shown in Figure 3c. Normalization was performed on these 48 cropped images to obtain uniformity in size among the images. In the same way, digitization and pre-processing were performed on all of the paper documents in the dataset. Finally, a dataset comprising a total of 24,000 word images was prepared.

### 3.3. Dataset Distribution in Respective Folders

In this, the cropped images belonging to a single class of the Gurumukhi month were separated from the images belonging to the other month classes. The same process was performed for all classes of the Gurumukhi months. Hence, after dataset sorting, the 24,000 total cropped images of the Gurumukhi month dataset were sorted according to 24 different classes (each class consisting of 1000 images) and saved into different folders.

### 3.4. Data Normalization

Image normalization was used to keep the CNN architectures numerically stable. The cropped word images, originally grayscale images, were normalized to a scale of 0–1 by multiplying each pixel value by 1/255. A model is supposed to learn faster when normalization is used.

### 3.5. Data Augmentation

Data augmentation techniques were used to boost the quantity of the cropped images. Different transformation techniques, such as rotation, shifting, shearing, and zooming, were used to supplement the existing data.

## 4. Methodology

Pre-trained networks or transfer learning models have been widely proposed for image classification problems on limited datasets. While training on these small datasets, these models provide remarkable benefits related to a variety of issues, such as model overfitting, etc. Along with these benefits, the use of transfer learning models sometimes faces various limitations, such as negative transfer, small resultant parameters, the need for higher processing speed, inaccurate identification of decision boundaries among multiple classes of dataset in the target domain, etc. As a result, they are unsuitable for real-time applications like automatic month name recognition systems for regional languages.

In the present work, the authors evaluated the performance of the most promising transfer learning models, ResNet 50, VGG19, and VGG16, on a dataset consisting of handwritten words. It was observed that the performance of these models has not been widely accepted in tasks such as the classification of a dataset comprising handwritten Gurumukhi words into 24 different classes. The results obtained using these transfer learning models are presented in Section 6.

Furthermore, the authors prepared a large dataset of over 24,000 images of words for the present research work. Hence, a CNN model could be developed from scratch for the given classification problem, as the dataset has a sufficient number of samples to perform both training and testing of the model. This will also help to improve the classification accuracy of the Gurumukhi handwritten word dataset.

In general, a convolutional neural network is a multiple-layer trained model connected in an end-to-end manner. A typical CNN architecture consists of a series of layers. Convolutional layers and pooling layers are two of its initial layers. These layers perform the majority of the computation in the CNN network. The mathematical expression for convolution is given in Equation (1), where the input array is represented by *f*, the kernel or filter by *h*, and the indexes of rows and columns in the resultant matrix by *m* and *n*.

(1) $$G\left[m,n\right]=\left(f*h\right)\left[m,n\right]=\underset{j}{\sum }\underset{k}{\sum }h\left[j,k\right]f\left[m-j,n-k\right]\;$$

Even though a convolutional layer’s role is to identify possible conjunctions of features from the previous layer, the pooling layer’s role is to combine semantically similar features into one. As a result, pooling reduces the spatial dimensions of representation. For example, let us say that the pooling layer accepts the input volume sizes W_ip_, H_ip_, and D_ip_ before pooling (where W_ip_ represents input width, H_ip_ represents input height, and D_ip_ represents input depth); then, the output volume size after pooling is W_op_, H_op_, and D_op_ (where W_op_ represents output width, H_op_ represents output height, and D_ip_ represents output depth).

Wop = (Wip − F)/S + 1(2)

Hop = (Hip − F)/S + 1(3)

Dop = Dip (remain Unchanged)(4)

The feature hierarchy is built by the CNN network’s two interleaved main layers, convolutional and pooling, and is then transferred to several fully connected layers for network output, where the Softmax function is applied to calculate the training loss. This loss is scaled to the minimum value possible value using various appropriate means.

In the present work, the authors designed a convolutional neural network with five convolutional layers, three max-pooling layers, and one output layer. The present work, performed using the proposed CNN model design, is novel and unique, because a dataset for the problem of classifying word images into 24 Gurumukhi month classes was self-prepared by using 500 distinct writers. This dataset of handwritten Gurumukhi month names is not available either online or offline. In the experimental phase of this paper, the dataset was initially simulated using various transfer learning models named ResNet 50, VGG19, and VGG16, the results of which were not promising in terms of classification accuracy. Hence, in order to improve and validate the results on a custom dataset, a CNN model was built from scratch to classify the given word images into one of the 24 classes corresponding to Gurumukhi months. To choose the optimal values of the model’s training parameters, such as optimizer selection, learning rate, number of epochs, batch sizes, etc., the authors performed various trials for parameter value selection, along with rigorous analysis of the results.

### 4.1. Proposed CNN Model

**Figure 4:**
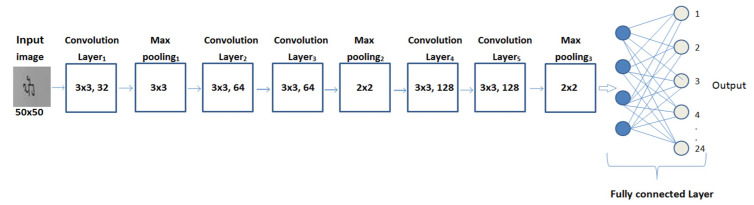
Architecture of proposed CNN model.

The proposed model is intended to classify a given word image into one of the 24 classes corresponding to Gurumukhi months, and a detailed description of it is given in the following section. For the classification of 24 different classes of Gurumukhi months, a new CNN-based model is proposed, the architecture of which is given in Figure 4.

The proposed model has five convolutional layers, three max-pooling layers, and one output layer. The first convolutional layer of the model comprises 32 weight filters of size (3 × 3) applied on an image of size (50 × 50), yielding 32 feature maps. The resulting 32 feature maps obtained from the first convolutional layer are passed to the first max-pooling layer. The first max-pooling layer has a filter size of (3 × 3), resulting in 32 feature maps with a size of (16 × 16), which are passed to the second convolutional layer of the network. Both the second and third convolutional layers of the network comprise 64 weight filters in each of the layers, with the same filter size of (3 × 3). The output parameter of the third convolutional layer is 64 feature maps with a size of (16 × 16), which are fed to a second max-pooling layer, resulting in 64 feature maps with a size of (8 × 8). The output of the second max-pooling layer is fed into the fourth convolutional layer, which is composed of 128 weight filters with a size of (3 × 3).

From the fourth convolutional layer, the output data of 128 feature maps of (8 × 8) size is fed through the fifth convolutional layer, which has the same number of filters as well as filter size as the fourth convolutional layer. The output of the fifth convolutional layer is then routed through the third max-pooling layer, yielding 128 feature maps with a size of (4 × 4). In the end, the final features from all these layers are passed to a fully connected layer that employs the Softmax activation function. From the fully connected layer, these features are transferred into the 24 classes of Gurumukhi months. Hence, the designed CNN model is able to classify the word images into the corresponding class of Gurumukhi month name.

Table 2 shows detailed descriptions for filter size, number of filters, input image size, output image size, and number of parameters used for each layer.

### 4.2. Description of Bilinear Model of CNN

**Figure 5:**
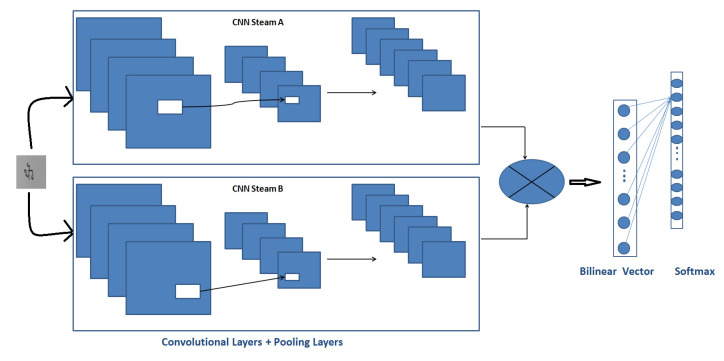
Architecture of bilinear CNN.

Lin et al. [30] created the first bilinear model. It was developed for fine-grained classification, detection and recognition tasks. Bilinear CNN is composed of two branches of CNN. These two branches of CNN work as feature extractors, whose output vectors are pooled bilinearly via an outer product function. Hence, compared to general CNN, the BCNN model generates a large amount of information. The network diagram for it is shown in Figure 5.

B-CCN employs a two-way convolutional neural network represented by CNN stream A and CNN stream B. They extract two features from each position in the image, multiply the outer product, and finally proceed with classification by the classification layer. CNN stream A locates the feature region of the image, and CNN stream B extracts the features from CNN A’s detected feature region [31]. As a result of this, the image classification process’s local detection and feature extraction tasks have been completed.

### 4.3. Proposed Model’s Training Parameters

The parameters selected for the proposed CNN model are presented in Table 3. The unified parameters of the proposed model presented in Table 3 include detailed information about the selected optimizer, learning rate, loss function, matrix, number of epochs, and batch sizes.

While selecting an appropriate optimizer for the proposed CNN model, performance benchmarking was conducted by simulating the proposed CNN model using different optimizers, including stochastic gradient descent (SGD), Adagrad, Adadelta, RMSprop, Nadam, and Adam. It was determined that model performance was decreased by 0.37%, 15.42%, 74.92%, 0.08%, and 0.2% when using SGD, Adagrad, Adadelta, RMSprop, and Nadam, respectively. The Adam optimizer outperformed the other optimizers in terms of accuracy, and hence it was chosen.

The most basic and widely used metric for evaluating CNN models is accuracy, but precision, recall, and F1 score are also required for assessing the quality of a model. Hence, all of these parameters were chosen in the present work to assess the performance of the proposed model.

The proposed model was initially simulated at the highest learning rate. It was discovered that the best results could be found at a learning rate of 0.0001 in terms of accuracy, but the results were not stable at higher learning rates than this.

While training the proposed model at different epochs (100 and 40) and batch sizes (20, 30, and 40), it was observed that the proposed model achieved its maximum accuracy at 100 epochs and a batch of size 20. Hence, the same training parameters have been chosen to simulate the CNN model on custom dataset.

## 5. Experiments and Result Analysis

This section contains the detailed results of the various experiments performed on the dataset of Gurumukhi months using the proposed model, along with an analysis of those results. For the classification of the word images in the Gurumukhi month dataset, the proposed model was run at different epochs and batch sizes. An analysis of the performance of the proposed model in terms of precision, recall, accuracy and F1 score is presented below.

### 5.1. Simulation of Proposed Model at 100 Epochs with Different Batch Sizes

The proposed CNN model was evaluated using a prepared dataset of Gurumukhi months for 100 epochs with batch sizes of 20, 30, and 40. The dataset split of 80% for training and 20% for testing remained consistent when running the model across all different batch sizes.

#### 5.1.1. Analysis with Batch Size 20

**Figure 6:**
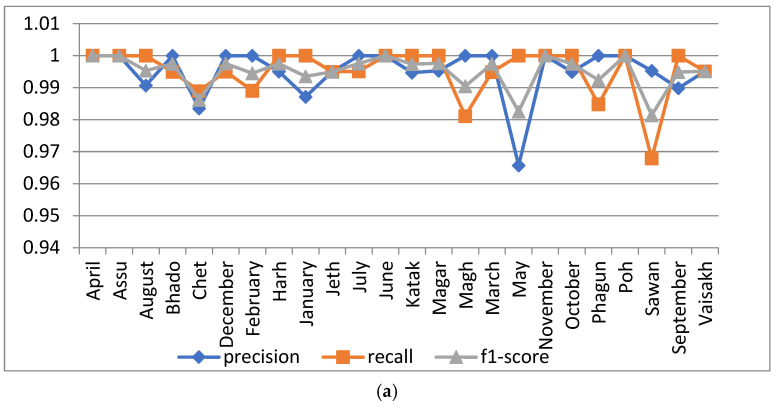

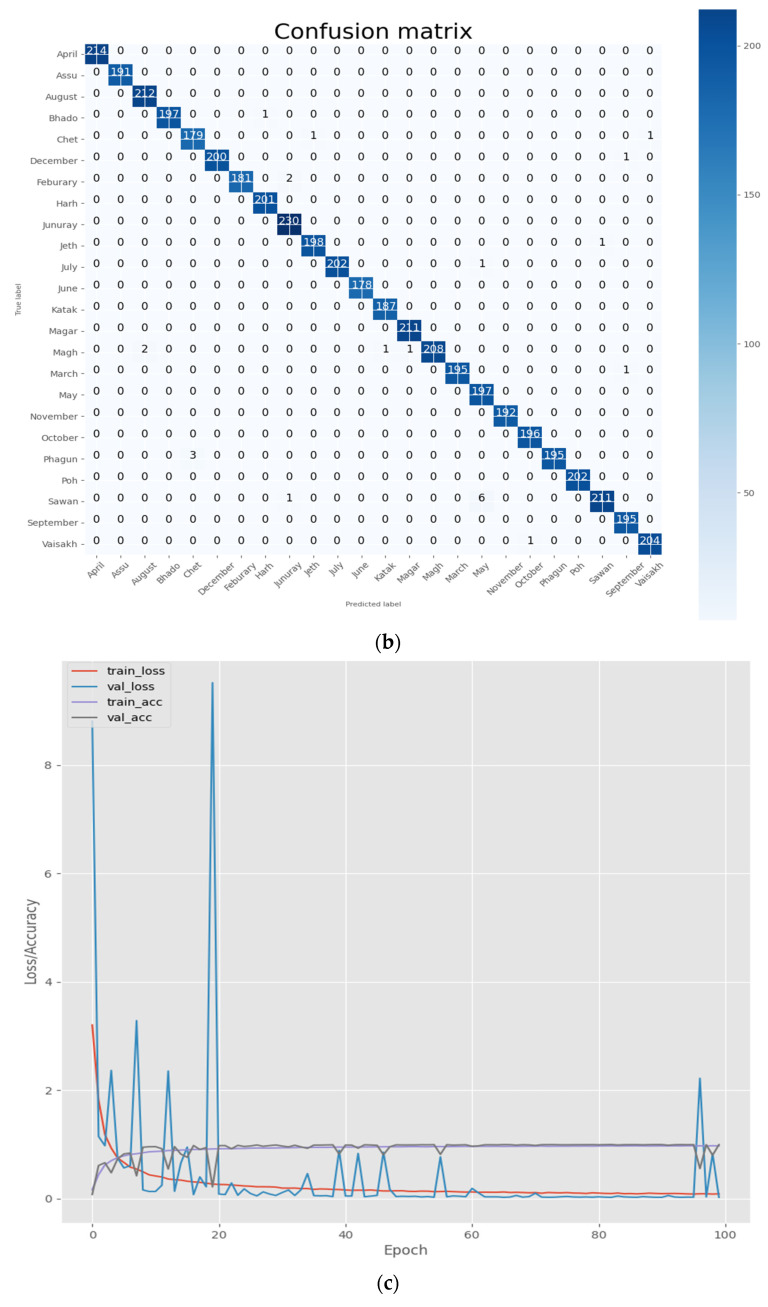
Analysis of the proposed model at 100 epochs and a batch size of 20: (**a**) precision, recall and F1 score of the 24 classes; (**b**) confusion matrix; (**c**) accuracy and loss curve.

The proposed model was simulated with 100 epochs and a batch size of 20. Its performance was analyzed in terms of precision, recall and F1 score with respect to the 24 dataset classes, as shown in Figure 6a. From the Figure, it can be observed that the resulting precision was is 1 for the ‘April’, ‘Assu’, ‘Bhado’, ‘December’, ‘February’, ‘July’, ‘June’, ‘Magh’, ‘March’, ‘November’ ‘Phagun’ and ‘Poh’ month class names. A minimum precision value of 0.9656 was oserved for the month ‘May’, and a minimum F1score of 0.9813 was observed for ‘Sawan’. The confusion matrix of the proposed model for 100 epochs with a batch size of 20 is shown in Figure 6b.

In Figure 6c, the accuracy and loss curves of the proposed model are presented. As per the curve, the training and validation accuracy of the proposed model was 97.03% and 99.50%, respectively, when simulated for 100 epochs with a batch size of 20.

#### 5.1.2. Analysis with Batch Size 30

**Figure 7:**
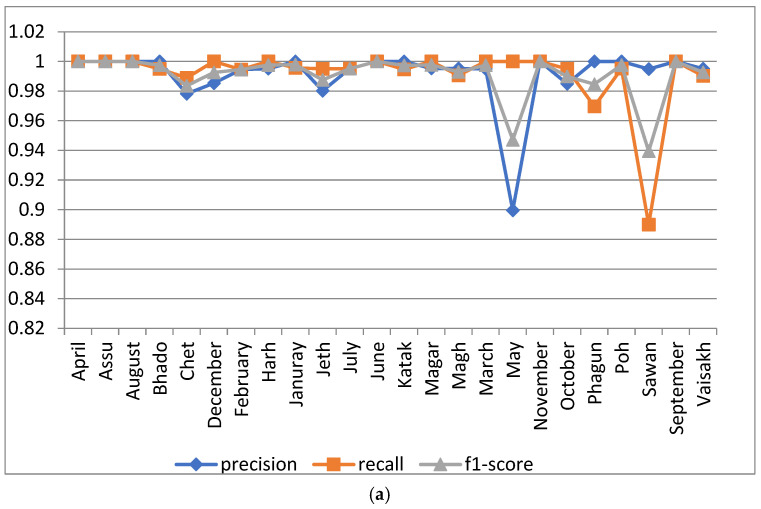

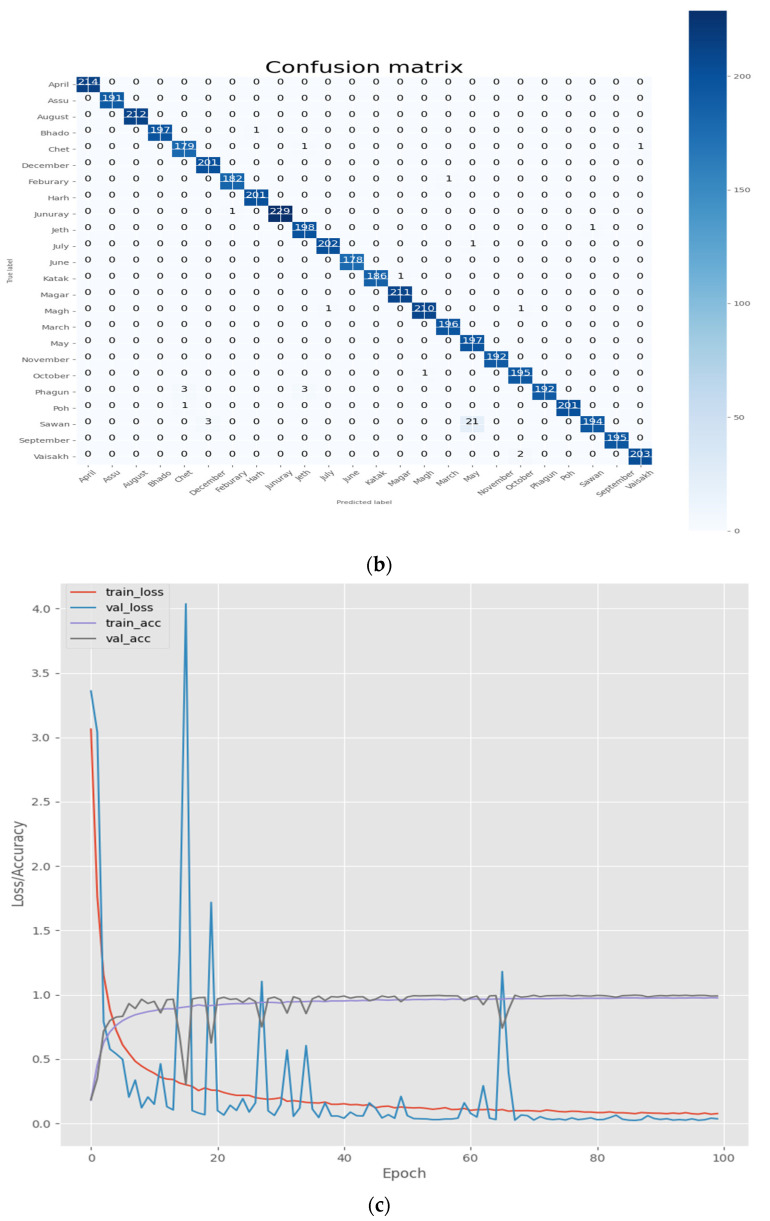
Analysis of proposed model at 100 epochs and a batch size of 30: (**a**) precision, recall, and F1 score for the 24 classes; (**b**) confusion matrix; (**c**) accuracy and loss curve.

For this analysis, the proposed model was run using 100 epochs and a batch size of 30. The various results of the proposed model with these parameters in terms of precision, recall and F1 score are shown in Figure 7a. As per the figure, the resulting precision value of the proposed model is one for the class name ‘April’, ‘Assu’, ‘August’, ‘Bhado’, ‘January’, ‘June’, ‘Katak’, ‘November’, ‘Phagun’, ‘Poh’, and ‘September’. For the month of ‘May’, the precision has a minimum value of 0.8995, shown in Figure 7a. The F1 score is at its minimum in the case of the class name ‘Sawan’, with a value of 0.9394. The confusion matrix of the proposed model for 100 epochs and a batch size of 30 is shown in Figure 7b.

Figure 7c, in this section, presents the training and validation accuracy results of the proposed model on 100 epochs and a batch size of 30. According to the graph, the training and validation accuracy of the proposed model with these specified parameters of epoch and batch size were around 97.63% and 99.08%, respectively.

#### 5.1.3. Analysis with Batch Size 40

**Figure 8:**
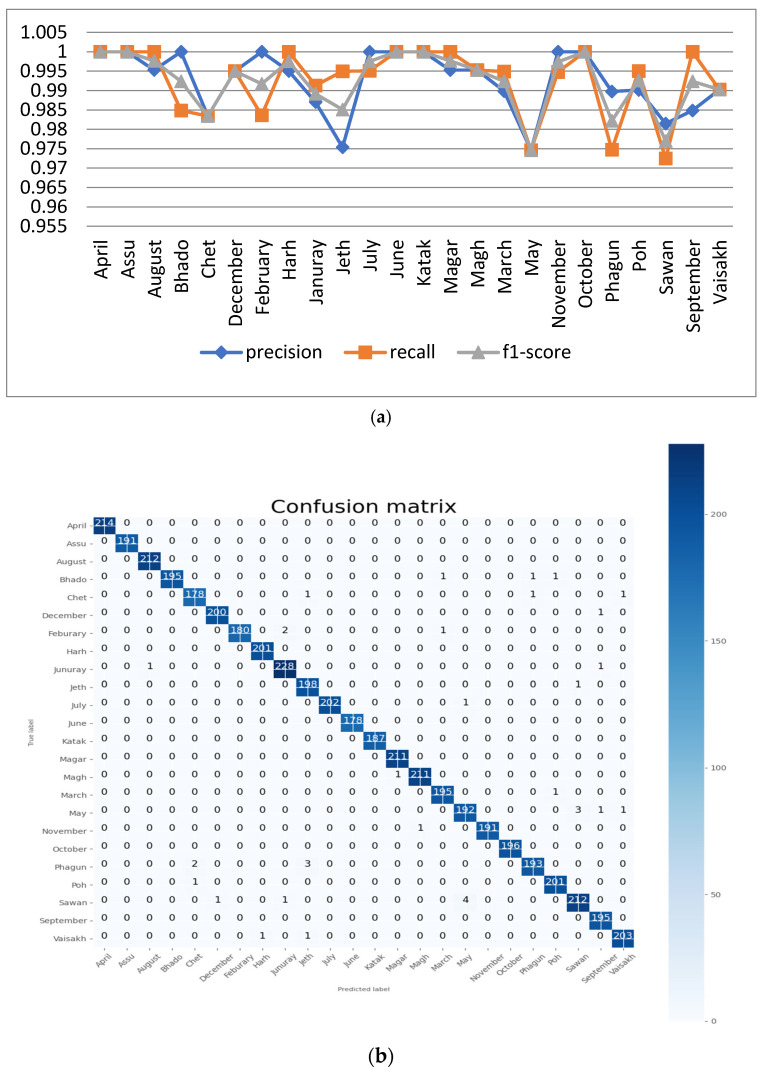

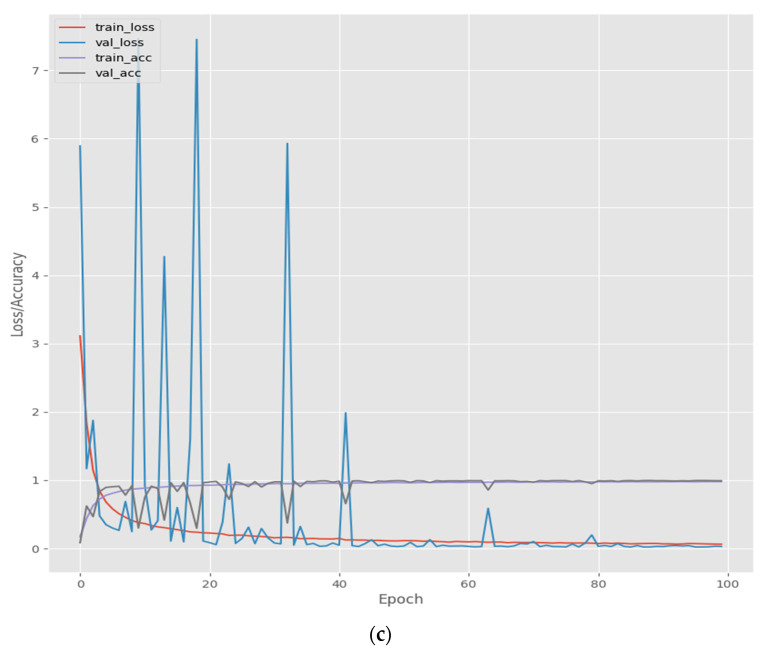
Analysis of proposed model at 100 epochs and a batch size of 40: (**a**) precision, recall, and F1 score for the 24 classes; (**b**) confusion matrix; (**c**) accuracy and loss curve.

For this experiment, the proposed CNN model was run at 100 epochs with a batch size of 40 batch size. The resulting performance analyses of the proposed model are shown in Figure 8a. It can be seen from the figure that the value of precision is 1 for the month names ‘April’, ‘Assu’, ‘Bhado’, ‘February’ ‘July’, ‘June’, ‘Katak’, ‘November’ and ‘October’. The minimum precision value and F1 score is 0.9746, which is for the month ‘May’. The confusion matrix of the proposed model at 100 epochs and a batch size of 40 is shown in Figure 8b.

The accuracy and loss results of this experiment are shown in Figure 8c. It can be seen from the figure that, for 100 epochs and a batch size of 40, the proposed model achieved training and validation accuracy of around 98.07% and 99.25%, respectively.

### 5.2. Simulation of Proposed Model at 40 Epochs with Different Batch Sizes

The proposed CNN model was also put to the test using the dataset of Gurumukhi months for 40 epochs with batch sizes of 20, 30, and 40. When running across multiple batch sizes, the dataset was split, with 80% being used for training, and 20% for testing remained stable.

#### 5.2.1. Analysis with Batch Size 20

**Figure 9:**
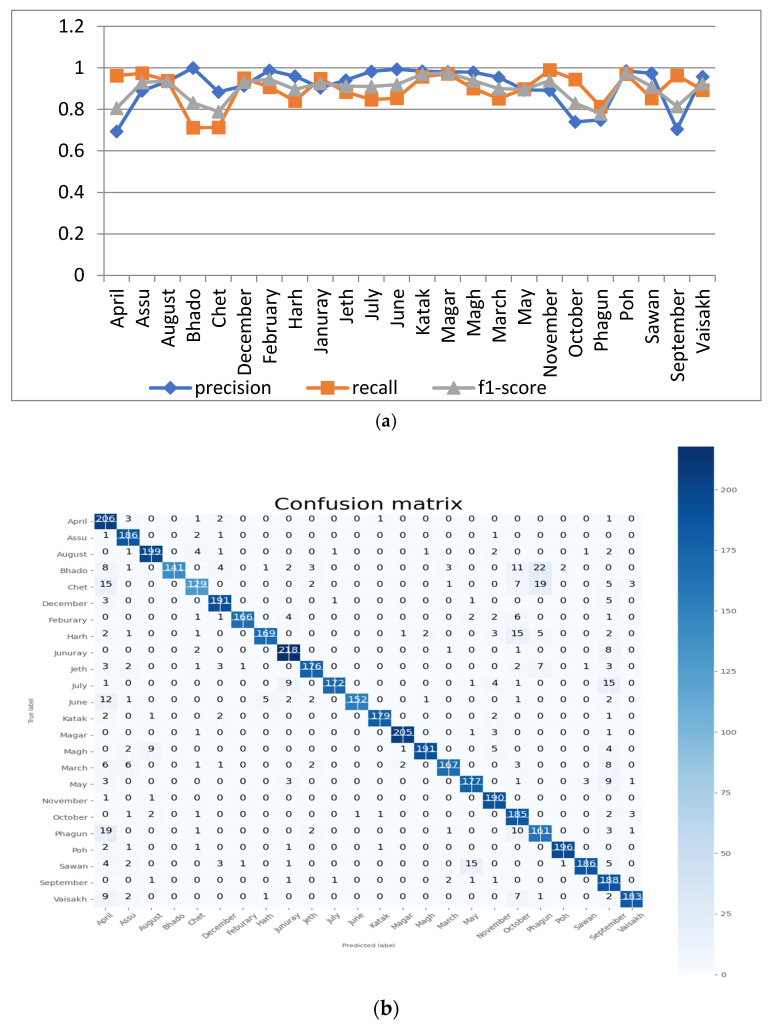

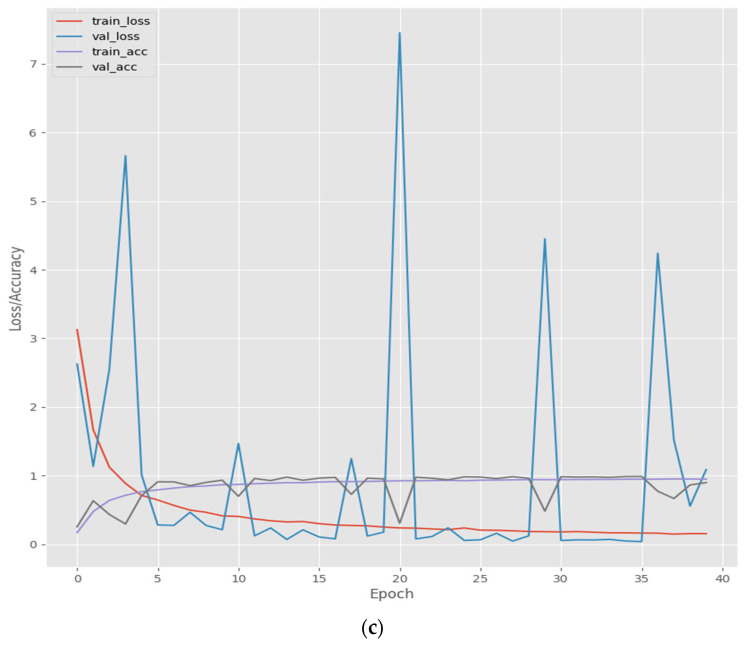
Analysis of the proposed model at 40 epochs and a batch size of 20: (**a**) precision, recall and F1 score for the 24 classes; (**b**) confusion matrix; (**c**) accuracy and loss curve.

The proposed model was tested for 40 epochs with a batch size of 20 in this analysis. For the aforementioned parameters of epoch number and batch size, analyses of the proposed model’s performance are depicted in Figure 9a in terms of precision, recall and F1 score for all 24 different classes represented in the dataset. The figure shows that the resultant value of precision is 1 for the month name ‘Bhado’, and it has a minimum value of 0.6936 for the month name ‘April’. The F1 score has a minimum value of 0.7796 for the month name ‘Phagun’. The confusion matrix of the proposed model at 40 epochs and a batch size of 20 is shown in Figure 9b.

The accuracy and loss curves of the proposed model are shown in Figure 9c. It can be observed from the figure that the training and validation accuracies of the proposed model with 40 epochs and a batch size of 20 are around 94.86% and 89.85%, respectively.

#### 5.2.2. Analysis with Batch Size 30

**Figure 10:**
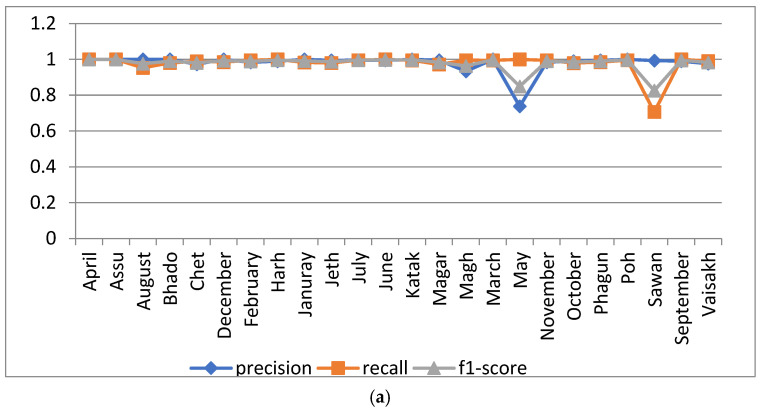

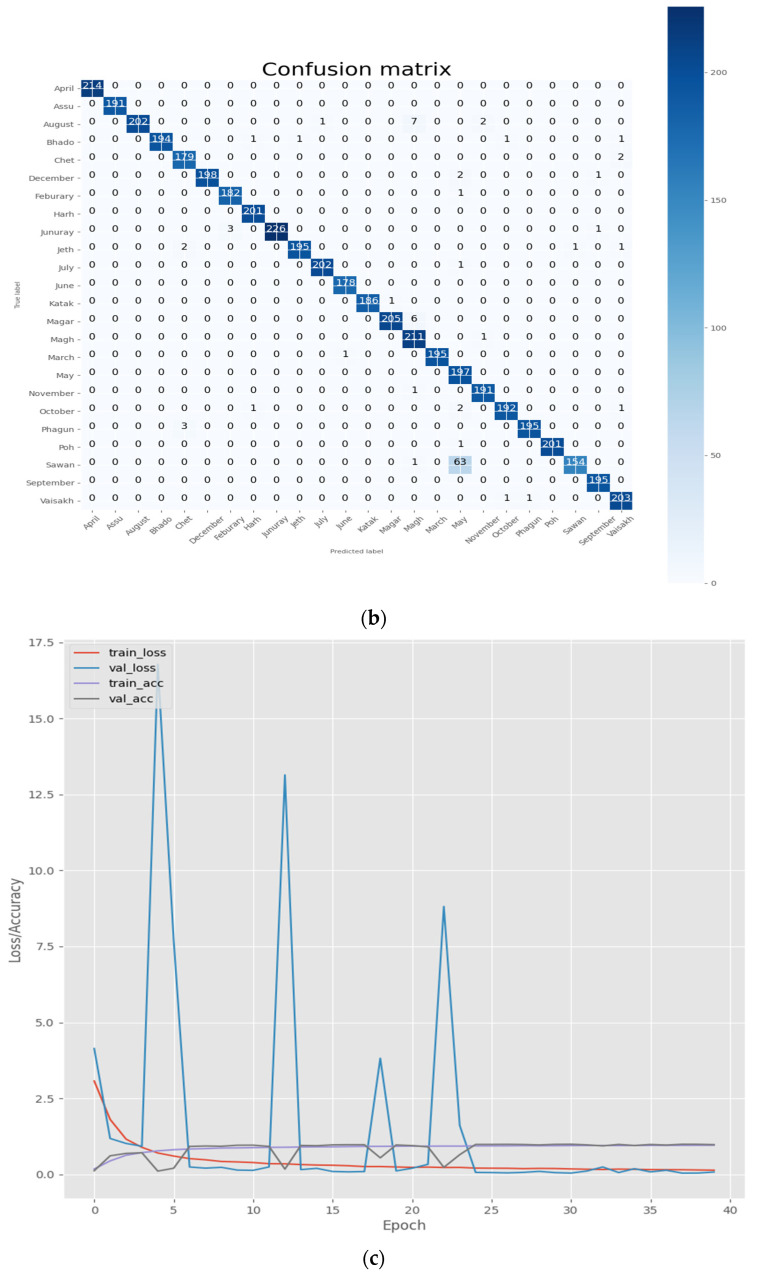
Analysis of proposed model at epochs 40 and batch size 30 (**a**) precision, recall and f1 score of 24 classes (**b**) confusion matrix (**c**) accuracy and loss curve.

The batch size of the proposed model was changed to 30 for simulation at 40 epochs. The resultant performance analysis of the proposed model in terms of various performance parameters is shown in Figure 10a. The figure indicates that the precision value for the proposed model was 1 for a number month classes, including ‘April’, ‘Assu’, ‘August’, ‘Bhado’, ‘December’, ‘January’, ‘Katak’, ‘March’, and ‘Poh’. The minimum value of precision was 0.7378 in the case of the month ‘May’. The confusion matrix of the proposed model at 40 epochs and a batch size of 30 is shown in Figure 10b.

In Figure 10c, the accuracy and loss of the proposed model are presented. According to the accuracy curve, the proposed model achieved maximum training and tested accuracies of around 95.50% and 97.65% at 40 epochs with a batch size of 30.

#### 5.2.3. Analysis with Batch Size 40

**Figure 11:**
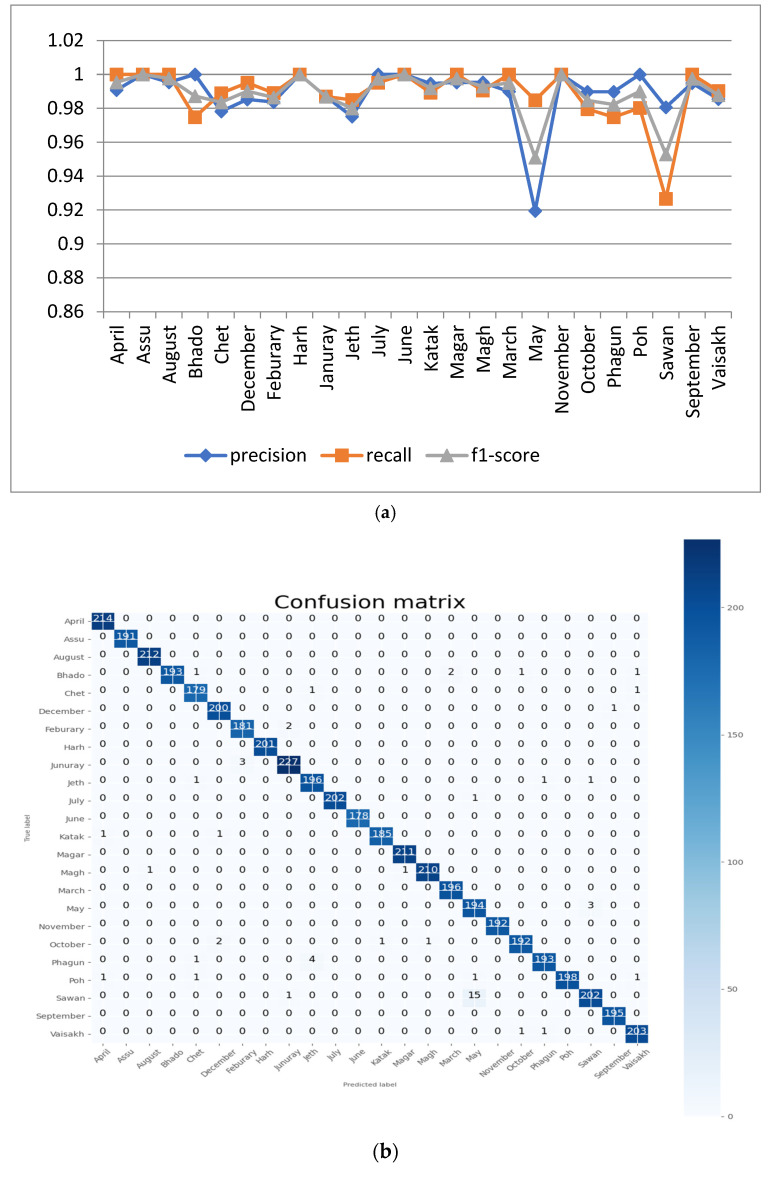

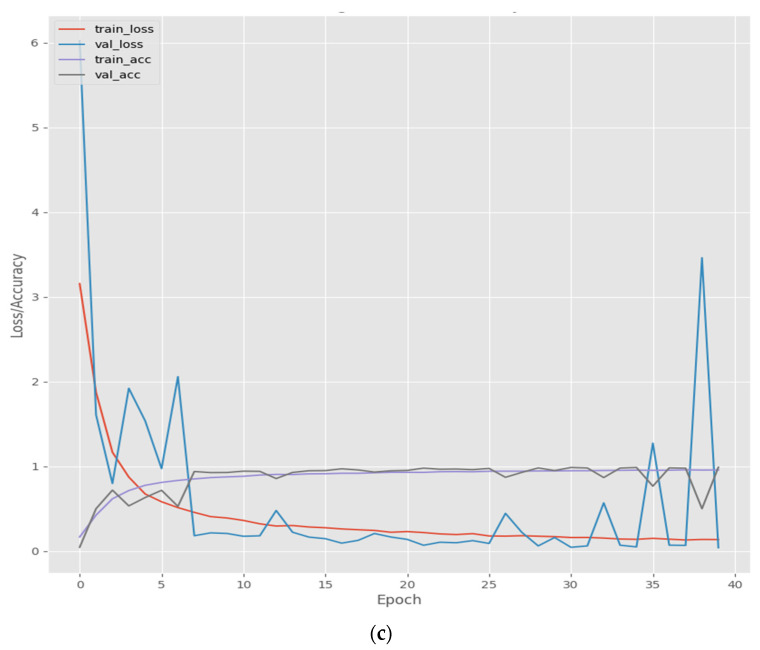
Analysis of the proposed model at 40 epochs with a batch size of 40: (**a**) precision, recall and F1 score of 24 classes; (**b**) confusion matrix; (**c**) accuracy and loss curve.

In this experiment, the model was run with a batch size of 40 at 40 epochs. The performance analysis of the proposed model is shown in Figure 11a. According to the figure, the model obtained a precision value of 1 for ‘Assu’, ‘Bhado’, ‘Harh’, ‘July’, ‘June’, ‘November’ and ‘Poh’ months, and had a minimum precision value of 0.9194 for the month of ‘May’. The confusion matrix of the proposed model at 40 epochs with a batch size of 40 is shown in Figure 11b.

In Figure 11c, the accuracy curve for the proposed model shows that the maximum training and validation accuracies were 95.68% and 98.85%, respectively, when the model ran for 40 epochs with a batch size of 40.

### 5.3. Analysis of Proposed Model with Different Numbers of Epochs and Different Batch Sizes

**Figure 12:**
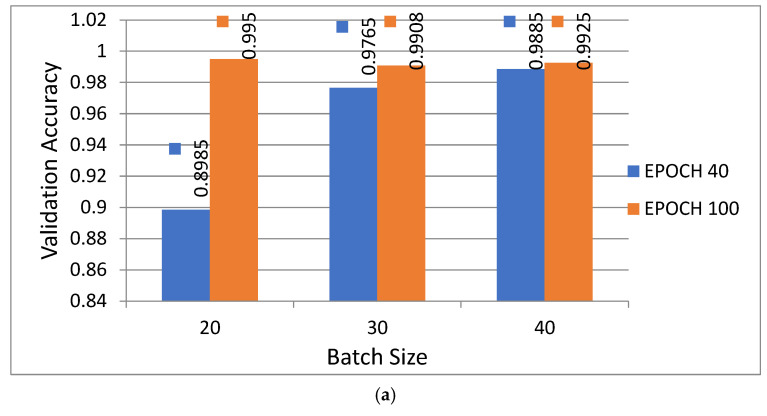

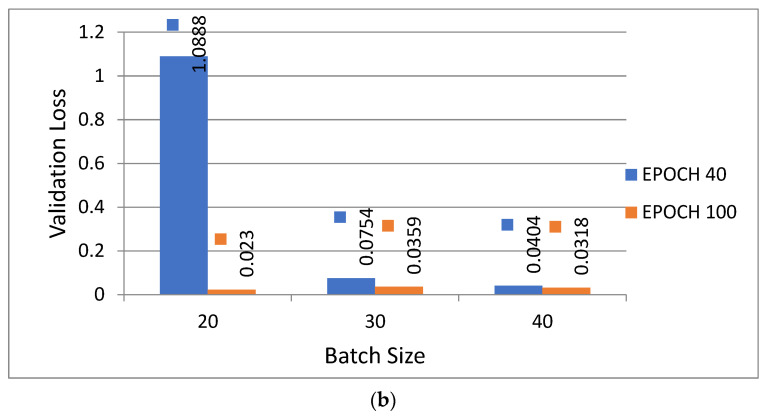
Performance of proposed model at 100 and 40 epochs with batch sizes of 20, 30 and 40: (**a**) validation accuracy graph; (**b**) validation loss graph.

An experiment was performed to evaluate the effectiveness of the proposed model using different numbers of epochs and different batch sizes. Figure 12a shows a comparative performance analysis for the proposed model with two different numbers of epochs, 100 and 40, and with batch sizes of 20, 30 and 40 in terms of validation accuracy, while Figure 12b shows validation loss in the form of a 2D column graph. Figure 12a,b show that the proposed model achieved the best results in terms of having the highest validation accuracy and the minimum validation loss at after 100 epochs with a batch size of 20, which is highlighted in orange color. It is also clear from the figures that the proposed model performs worst in the case with 40 epochs and a batch size of 20, which is highlighted with blue color.

## 6. Comparison of Proposed CNN Model with Transfer Learning Models at 100 Epochs and a Batch Size of 20

In this section, a comparative analysis of the proposed CNN model was performed with various transfer learning models, ResNet 50, VGG 19 and VGG16, with 100 epochs and a batch size of 20. In Table 4, below, a comparative analysis of the proposed model with transfer learning models in terms of training accuracy, validation accuracy, overall precision, overall recall, and overall F1 score is presented.

From Table 4, it can been found that the proposed model outperformed the transfer learning models ResNet 50, VGG19, and VGG16 in terms of results of training parameters as well as the results of confusion matrix parameters when simulated at 100 epochs and a batch size of 20 on a custom dataset of Gurumukhi handwritten month names.

## 7. Comparison of the Proposed Model against Existing Text Recognition Systems

To demonstrate the astonishing performance of the proposed CNN model in text recognition, a comparison against existing text recognition systems was carried out, and is reported in the present section.

As shown in Table 5, the proposed CNN model was compared against existing text recognition systems on the basis of the dataset, feature extraction method, and the selection of a classifier for a given classification problems.

As can be observed in Table 5, for a given classification problem, in the present work, the proposed CNN model achieved a recognition rate of 99.25%, which is the highest accuracy reported among the classification systems used for text recognition.

In addition, the dataset used for the present classification task is unique and new, and is not available either online or offline.

Furthermore, the number of training and testing samples in the present dataset is far greater than the number of training and testing samples used for text recognition by the existing models.

## 8. Conclusions

A CNN-based model was proposed for word recognition using a holistic approach. The proposed CNN model was designed with five convolutional layers, three pooling layers, and one fully connected layer. The experiment was validated using a self-prepared dataset of Gurumukhi months with 24,000 images of words in it. The proposed model was used for the simulation using various epochs and batch sizes. The performance for different numbers of epochs with the same batch size was compared, as was the same number of epochs with different batch sizes. The results of performance analysis reveal that the proposed model achieves its maximum validation accuracy with 100 epochs and a batch size of 20, and is around 99.50%. In the future, the authors will seek to develop and analyze the proposed model using different optimizers.

## Figures and Tables

**Table 1:** Detail overview of dataset.

Sr No.	Class Name in English	Class Name in Gurumukhi	Time Duration	Type of Month
1.	Vaisakh	ਵਿਸਾਖ	14 April to 14 May	Desi Months
2.	Jeth	ਜੇਠ	15 May to 14 June	
3.	Harh	ਹਾੜ੍ਹ	15 June to 15 July	
4.	Sawan	ਸਾਉਣ	16 July to 15 August	
5.	Bhado	ਭਾਦੋਂ	16 August to 14 September	
6.	Assu	ਅੱਸੂ	15 September to 14 October	
7.	Katak	ਕੱਤਕ	15 October to 13 November	
8.	Magar	ਮੱਘਰ	14 November to 13 December	
9.	Poh	ਪੋਹ	14 December to 12 January	
10.	Magh	ਮਾਘ	13 January to 11 February	
11.	Phagun	ਫੱਗਣ	12 February to 13 March	
12.	Chet	ਚੇਤ	14 March to 13 April	
13.	January	ਜਨਵਰੀ	1 January to 31 January	English Months
14.	February	ਫਰਵਰੀ	1 February to 28/29 February	
15.	March	ਮਾਰਚ	1 March to 31 March	
16.	April	ਅਪ੍ਰੈਲ	1 April to 30 April	
17.	May	ਮਈ	1 May to 31 May	
18.	June	ਜੂਨ	1 June to 30 June	
19.	July	ਜੁਲਾਈ	1 July to 31 July	
20.	August	ਅਗਸਤ	1 August to 31 August	
21.	September	ਸਤੰਬਰ	1 September to 30 September	
22.	October	ਅਕਤੂਬਰ	1 October to 31 October	
23.	November	ਨਵੰਬਰ	1 November to 30 November	
24.	December	ਦਸੰਬਰ	1 December to 31 December	

**Table 2:** Details of layers of proposed CNN model.

S.No.	Layers	Input Image Size	Filter Size	No. of Filter	Activation Function	Output	Parameters
1	Input Image	50 × 50 × 1	-----	-----	-----	-----	-----
2	Convolutional	50 × 50 × 1	3 × 3	32	ReLU	50 × 50 × 32	320
3	Maxpooling	50 × 50 × 32	Poolsize (3 × 3)	------	------	16 × 16 × 32	0
4	Convolutional	16 × 16 × 32	3 × 3	64	ReLU	16 × 16 × 64	18,496
5	Convolutional	16 × 16 × 64	3 × 3	64	ReLU	16 × 16 × 64	36,928
6	Maxpooling	16 × 16 × 64	Pool size 2 × 2	------	------	8 × 8 × 64	0
7	Convolutional	8 × 8 × 64	3 × 3	128	ReLU	8 × 8 × 128	73,856
8	Convolutional	8 × 8 × 128	3 × 3	128	ReLU	8 × 8 × 128	147,584
9	Maxpooling	8 × 8 × 128	Pool size 2 × 2	------	------	4 × 4 × 128	0
10	Flatten	4 × 4 × 128	----	-----	-----	2048	0
11	Dense	2048	----	-----	ReLU	1024	2,098,176
12	Dense	1024	----	-----	Softmax	24	24,600

**Table 3:** Proposed model’s training parameters.

Adam Optimizer’s Specification	Learning Rate (LR)	Loss Function	Matrix	Number of Epochs	Batch Size (BS)
learning rate = 1.0 × 10^−3^, beta1 = 0.9, beta2 = 0.999, epsilon = 1.0 × 10^−7^, decay= learning rate/epochs	0.0001	Categorical cross entropy	Accuracy	100	20

**Table 4:** Comparison of models.

		Training				Confusion Matrix		
	Parameters	Training	Validation	Training	Validation	Overall	Overall	Overall
Model		Accuracy	Accuracy	Loss	Loss	Precision	Recall	F1 Score
**ResNet 50**		0.3299	0.3929	2.1693	1.9268	0.4482	0.3937	0.3892
**VGG 19**		0.7530	0.7771	0.7560	0.6647	0.7929	0.7767	0.7756
**VGG 16**		0.7925	0.8138	0.6274	0.5484	0.8223	0.8135	0.8115
**Proposed Model**		**0.9703**	**0.9950**	**0.0885**	**0.0230**	**0.9950**	**0.9951**	**0.9950**

**Table 5:** Comparison of proposed model with existing text recognition systems.

The Authors (Year)	Technique Used		Dataset Used	Accuracy
	Feature Extraction Method	Classifier		
[12]	Parabola curve fitting and power curve fitting	SVM and k-NN	3500 offline handwritten Gurumukhi characters	98.10%
[15]	Discrete wavelet transforms, discrete cosine transforms, fast Fourier transforms and fan beam transforms	SVM	10,500 samples of isolated offline handwritten Gurumukhi characters.	95.8%
[17]	Local binary pattern (LBP) features, directional features, and regional features	Deep neural network	2700 images of Gurumukhi text	99.3%
[19]	Histogram oriented gradient (HOG) and pyramid histogram oriented gradient (PHOG) features	SVM	3500 handwritten Gurumukhi characters	99.1%
[20]	Zoning, diagonal, transition, intersection and open end points, centroid, the horizontal peak extent, the vertical peak extent, parabola curve fitting, and power curve fitting-based features	Naive Bayes (NB), decision Tree (DT), random forest (RF) and AdaBoostM1	49,000 samples of Gurumukhi handwritten text	89.85%
[22]	Zoning, discrete cosine transforms and gradient features	k-NN, SVM, decision tree (DT), random forest (RF)	Medieval HandwrittenGurumukhi Manuscripts	95.91%
[23]	Zoning, diagonal, peak extent-based features (horizontally and vertically) and shadow features	k-NN, decision tree (DT) and random forest	8960 samples of Gurumukhi handwritten text	96.03%
[25]	Vertically peak extent, diagonal, centroid features	k-NN, linear- (SVM), RBF-SVM, naive Bayes, decision tree, CNN, and random forest	13,000 samples that includes 7000 characters and 6000 numerals.	87.9%
[26]	Automatic feature extraction	Convolutional neural network	3500 Gurumukhi characters	98.32%
**Proposed Model**	**Automatic feature extraction**	**Convolutional neural network**	**24,000 Gurumukhi Month Name Images**	**99.50%**

## Data Availability

The data that supports the findings of this paper is available from the first author upon reasonable request.
